# Newly Discovered Cutting-Edge Triple Combination Cystic Fibrosis Therapy: A Systematic Review

**DOI:** 10.7759/cureus.29359

**Published:** 2022-09-20

**Authors:** Sarah N Dawood, Ahmad M Rabih, Ahmad Niaj, Aishwarya Raman, Manish Uprety, Maria Jose Calero, Maria Resah B Villanueva, Narges Joshaghani, Nicole Villa, Omar Badla, Raman Goit, Samia E Saddik, Lubna Mohammed

**Affiliations:** 1 Pediatrics, California Institute of Behavioral Neurosciences & Psychology, Fairfield, USA; 2 Internal Medicine, California Institute of Behavioral Neurosciences & Psychology, Fairfield, USA; 3 Obstetrics and Gynecology, California Institute of Behavioral Neurosciences & Psychology, Fairfield, USA; 4 Research, California Institute of Behavioral Neurosciences & Psychology, Fairfield, USA; 5 Psychiatry and Behavioral Sciences, California Institute of Behavioral Neurosciences & Psychology, Fairfield, USA; 6 General Surgery, California Institute of Behavioral Neurosciences & Psychology, Fairfield, USA

**Keywords:** cftr gene mutation, phe508 mutation, cftr modulator, ivacaftor, tezacaftor, elexacaftor, cftr modulating therapy, trikafta, cystic fibrosis (cf), cystic fibrosis triple therapy

## Abstract

A cystic fibrosis (CF) transmembrane conductor regulator (CFTR) gene modulating triple therapy combining elexacaftor-tezacaftor-ivacaftor (Trikafta) has been recently discovered. Its approval by the Food and Drug Administration (FDA) in 2019 has expanded the target therapy group to individuals aged twelve and up with at least one Phe508del (phenylalanine 508 deletion) mutation in the CFTR gene. This systematic review aims to assess this combination therapy's safety and efficacy. Following the Preferred Reporting Items for Systematic Reviews and Meta-Analyses (PRISMA) 2020 guidelines, an in-depth search was performed. The search was done by utilizing databases such as PubMed Central (PMC), Google Scholar, and Science Direct for articles related to this topic. Studies published in the last five years in the English language were chosen preliminarily. Further eligibility criteria and quality assessment tools were employed to assess the risk of bias and finalize ten articles to be used in this review. The chosen articles constituted four randomized control trials (RCTs), four systematic reviews, and two narrative reviews. The last date for data collection was April 24, 2022.

Based on the findings of this review, we concluded that by combining three CFTR modulators, this therapy had outperformed all the currently available medications in terms of improving pulmonary function, reducing exacerbations, and enhancing the quality of life of CF patients. In clinical trials, headache and rash were the most common side effects, and laboratory testing to assess liver function is suggested. Long-term safety and effectiveness must be confirmed by the continued review of real-life patient data. Studies done on triple therapy thus far have been promising. Unfortunately, a small proportion of the CF population remains ineligible for any form of CFTR modulator therapy owing to their type of genetic mutation, and this provides ground for further research in this field.

## Introduction and background

Inherited in an autosomal recessive (AR) pattern, cystic fibrosis (CF) is a progressive disease that affects roughly 80,000 children and adults worldwide [1]. The disease occurs due to mutations in the CF transmembrane conductor regulator (CFTR) gene, which leads to CFTR protein abnormalities. Typically, this protein encodes for an ion channel responsible for transporting bicarbonate and chloride ions across the cell membrane. In the case of mutant CFTR genes, there may be a decrease in protein quantity or an absence of activity [2]. The lack of functioning CFTR protein hinders the release of chloride and bicarbonate into airway secretions. This results in thickened secretions which subsequently allow bacterial entry, leading to recurrent respiratory infections. Respiratory failure caused by chronic mucosal inflammation and opportunistic microbial infection remains the primary cause of death in CF patients. Owing to a similar mechanism, exocrine pancreatic insufficiency and reproductive system diseases develop in CF patients leading to complications such as fat malabsorption and infertility, respectively [1].

**Figure 1:**
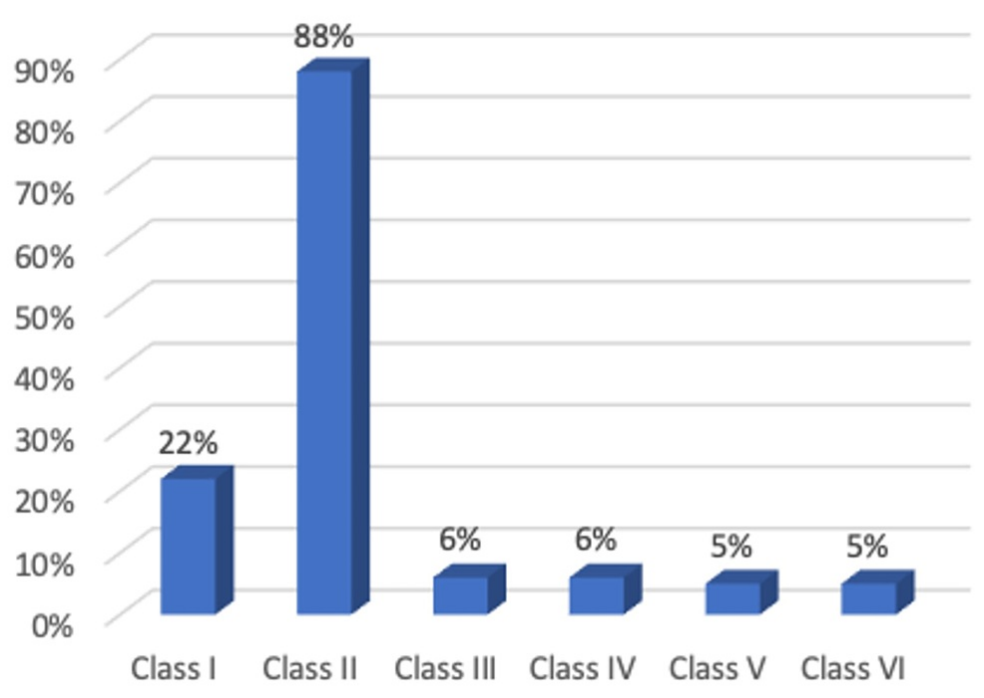
Prevalence of having at least one mutation from each class among cystic fibrosis patients

Over 2,000 variations of the CFTR gene exist, each of which affects the CFTR protein in terms of its function and production. Six different classes have been identified that describe the various mutations based on the mechanism of CFTR activity disruption (represented in Table 1 and Figure 1) [3]. This paper will focus on Class II mutations, as these are the most predominant ones globally. It commonly forms when the amino acid phenylalanine (Phe) gets deleted from the allele at location 508 (Phe508del). At least one copy of this mutation can be found in up to 90 percent of all CF patients, with over half being homozygous for Phe508del [4]. In patients with these mutations, the CFTR protein gets synthesized; however, it is misfolded, hindering its presentation to the cell surface [5]. These molecular defects need to be addressed to restore Phe508del CFTR function [6].

**Table 1:** The six classes of CFTR gene mutations Cystic fibrosis transmembrane conductor regulator (CFTR).

Mutation	Description
Class I	Absent functional CFTR
Class II	Misfolded CFTR protein is made; impaired transport to the cell surface
Class III	CFTR is made and reaches the surface, but the ion channel fails to open
Class IV	CFTR is made and reaches the surface, but channel function is impaired
Class V	Normal CFTR made and moves to the surface, but in inadequate amounts
Class VI	CFTR moves to the cell membrane but has reduced stability

Currently, nearly all CF therapies address the downstream consequences of CFTR abnormalities rather than the genetic issue at a cellular level. This includes conventional antibiotics, mucolytics, and anti-inflammatory agents. While these CF therapies that are currently used provide symptomatic relief for CF patients, they permit acceleration of the disease and lead to complications that may be hard to treat [1]. With recent breakthroughs in research, small compounds, known as CFTR modulators, have been produced that target the root of the problem by directly modifying the CFTR protein [3]. Based on their function, the CFTR modulators are classified into four types: potentiators, correctors, stabilizers, and amplifiers. The fact that these medicines are orally accessible gives it the advantage of restoring protein function systematically, which in turn prevents the disease from progressing to a fatal stage [4]. Combining lumacaftor or tezacaftor (CFTR correctors) with ivacaftor (CFTR potentiator) in patients homozygous for the Phe508del mutation have significantly improved lung function. However, these dual combinations have failed to improve lung function when administered to individuals with a single Phe508del allele and any other second mutation [6]. The efficacy of a new regimen composed of elexacaftor, tezacaftor, and ivacaftor was recently demonstrated in CF patients with no less than one Phe508del mutation. Spirometry, respiratory symptoms, and sweat chloride content have all improved significantly as a result of this treatment. Additionally, compared to the dual combination of tezacaftor and ivacaftor (TEZ/IVA), it has shown considerably greater efficacy in patients who are homozygous for Phe508del [7]. This regimen can directly and fundamentally correct mutated CFTR genes, opening up new possibilities for a more significant proportion of CF patients worldwide [8]. This systematic review aims to discuss the efficacy and safety of the new triple therapy Trikafta with the help of data from various clinical trials.

## Review

Methods

The Preferred Reporting Items for Systematic Reviews and Meta-Analyses (PRISMA) 2020 guidelines were followed in this systematic review [9].

Eligibility Criteria

Studies with the following criteria were selected as eligible for the review; studies written in English, human studies, and free full-text articles published in the last five years (2017-2022). Further inclusion criteria include children six years and above with CF treated with triple combination therapy (Elexacaftor - Ivacaftor - Tezacaftor). Articles that included children under six, paid articles, animal studies, case studies, and editorials were excluded. 

Search Strategy and Databases

The databases PubMed Central (PMC), Google Scholar, and Science Direct were explored for potential articles. Using the Boolean method, Medical Subject Heading (MeSH) terms were combined with keywords to identify all potentially relevant articles pertaining to the efficacy and safety of the triple combination CFTR modulators in treating CF in the pediatric age group. The keywords used to conduct the search were cystic fibrosis, CF, elexacaftor (ELX), tezacaftor (TEZ), ivacaftor (IVA), CF triple therapy, AND CFTR modulators. The details of the search strategy are listed in Table 2. The EndNote reference manager was used to group all the references and remove duplicates. Irrelevant studies were then excluded based on titles and abstracts. Following this, full-text papers were reviewed for further exclusion.

**Table 2:** The strategy of the database search with their respective filters Cystic fibrosis transmembrane conductor regulator (CFTR); Phenylalanine 508 deletion mutation (Phe508del); Delta phenylalanine 508 deletion mutation-cystic fibrosis transmembrane conductor regulator gene (deltaPhe508-CFTR); Cystic fibrosis (CF).

Database	Search Strategy	Filters Applied	Results
PubMed	Cystic Fibrosis OR ( "Cystic Fibrosis/drug therapy"[Majr] AND CFTR OR Phe508del OR deltaPhe508-CFTR OR ( "Cystic Fibrosis Transmembrane Conductance Regulator/antagonists and inhibitors"[Majr] OR "Cystic Fibrosis Transmembrane Conductance Regulator/drug effects"[Majr] OR "Cystic Fibrosis Transmembrane Conductance Regulator/genetics"[Majr] OR "Cystic Fibrosis Transmembrane Conductance Regulator/pharmacology"[Majr] OR "Cystic Fibrosis Transmembrane Conductance Regulator/therapeutic use"[Majr] ) AND tezacaftor OR ivacaftor OR elexacaftor OR "elexacaftor, tezacaftor, ivacaftor drug combination" [Majr]	Free full text, last five years, human, English, child: six years and above	93
Google Scholar	Keywords: ivacaftor, lumacaftor, elexacaftor, tezacaftor, CF triple therapy, CFTR modulators, CF therapy.	2017 - 2022	200
Science Direct	Keywords: CFTR modulators, cystic fibrosis triple therapy	2017-2022, open access and open archive	90

Risk of Bias in Individual Studies

Quality assessment was done for the full-text articles using tools specific to each type of study. Studies with a score of >70% were finalized to be used in the paper (Table 3).

**Table 3:** Details of the quality assessment and the tools used for the final articles accepted for this review Cochrane collaboration risk of bias tool (CCRBT); Randomized controlled trials (RCTs); Scale for the assessment of narrative review articles 2 (SANRA 2); Assessment of multiple systematic reviews 2 (AMSTAR 2).

Quality Assessment Tool	Type of Study	Total Score	Accepted Score (>70%)	Number of Accepted Studies (#)
CCRBT	RCTs	7	5	4; Harry GM et al. 2019 [3], PG Middleton et al. 2019 [6], Edith T et al. 2021 [7], David P et al. 2022 [10].
SANRA 2	Narrative Review	12	9	2; Tewkesbury et al. 2021 [4], Marika et al. 2021 [8].
AMSTAR 2	Systematic Review, Meta-Analysis	16	12	4; Jennifer et al. 2019 [2], Andrea et al. 2020 [11], Anielo et al. 2021 [12], Dagenais et al. 2021 [13].

Results

Study Selection and Quality Assessment

**Figure 2:**
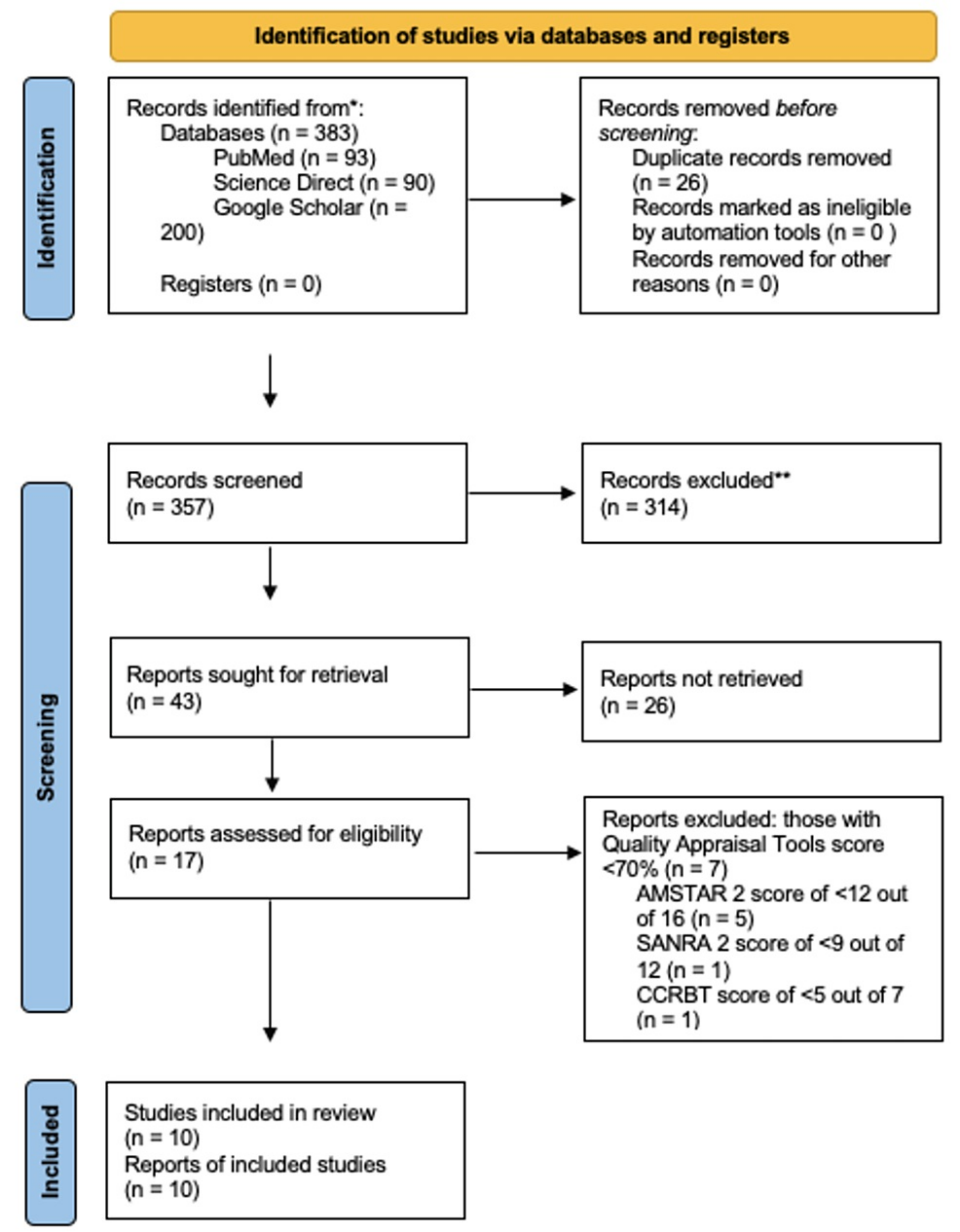
Prisma flow chart. Assessment of multiple systematic reviews 2 (AMSTAR 2); Scale for the assessment of narrative review articles 2 (SANRA 2); Cochrane collaboration risk of bias tool (CCRBT).

Using three databases (PubMed Central, Science Direct, and Google Scholar), a total of 383 results were obtained. These results were grouped to remove duplicates, which were 26 in number. The remaining 357 records were screened by title and abstract, and 314 irrelevant records were excluded. There were 43 reports left, which were thoroughly screened as full-text papers. Out of those, 26 were excluded. Quality assessment was then performed on the remaining studies, using tools specific to each type of study. This was done independently by the two authors, Sarah Dawood and Ahmad Rabih. 10 final studies with a score of >70% were included in this review. The studies constituted four randomized control trials (RCTs), four systematic reviews, and two narrative reviews. The last date for data collection was April 24, 2022. A flow diagram representing the search selection is shown in Figure 2.

Study Characteristics

The main characteristics of the randomized clinical trials are shown in Table 4.

**Table 4:** Main features of the randomized control trials chosen for this review Four final randomized control trials with similar inclusion criteria were chosen for this review and have been summarized in this table. Randomized control trial (RCT); Phenylalanine 508 deletion mutation (Phe508del); Percent predicted forced expiratory volume in one second (ppFEV1); Elexacaftor/Tezacaftor/Ivacaftor (ELX/TEZ/IVA); Tezacaftor/Ivacaftor (TEZ/IVA); Cystic fibrosis questionnaire–revised respiratory domain (CFQ-R RD); Cystic fibrosis (CF); Homozygous for the Phe508del-cystic fibrosis transmembrane conductance regulator mutation (F/F); Heterozygous for the Phe508del-cystic fibrosis transmembrane conductance regulator mutation and a minimal function cystic fibrosis transmembrane conductance regulator mutation (F/MF); Body mass index (BMI).

First Author, Year	Study Type	Inclusion Criteria	Sample Size (dropouts)	Intervention	Outcomes
Harry GM, 2019 [3]	RCT	Patients aged 12 years and above, homozygous for Phe508del mutation, with stable disease and ppFEV1 of 40 to 90	113 (6)	Participants were randomized 1:1 to four weeks of ELX/TEZ/IVA versus TEZ/IVA alone	The ELX/TEZ/IVA group showed improvements in ppFEV1, sweat chloride concentration, and CFQ-R RD score compared to the TEZ/IVA group. ELX/TEZ/IVA was well tolerated, with no discontinuations.
PG Middleton, 2019 [6]	RCT	Patients aged 12 years of age or older with CF and Phe508del– minimal function genotypes, ppFEV1 of 40 to 90% at screening, and had stable disease during the one-month screening period before the study began	403 (3)	Participants underwent randomization and received at least one dose of active treatment (ELZ/TEZ/IVA) or placebo	The ELZ/TEZ/IVA group resulted in a ppFEV1 that was 13.8 points higher at four weeks and 14.3 points higher through 24 weeks. The rate of pulmonary exacerbations was 63% lower, respiratory domain score on the CFQ-R RD 20.2 points higher, and a sweat chloride concentration 41.8 mmol per liter lower.
Edith T, 2021 [7]	RCT	Children aged 6 to 11 years, with CF, and either F/F or F/MF genotypes.	66 (2)	Children weighing <30 kg received 50% of the ELX/TEZ/IVA adult daily dose, whereas children weighing >30 kg received the total adult daily dose	ELX/TEZ/IVA treatment improved the ppFEV1, CFQ-R RD score, lung clearance index 2.5, and sweat chloride; body mass index-for-age z-score increased over the 24-week treatment period when compared with the pretreatment baseline.
David P, 2022 [10]	RCT	CF patients aged 12 years or older with at least one Phe508del allele starting ELZ/TEZ/IVA for the first time	487 (7)	Assessments occurred before and 1, 3, and 6 months into ELZ/TEZ/IVA therapy.	Six months into ELZ/TEZ/IVA therapy, ppFEV1 improved from baseline, CFQ-R RD score improved by 20.4 points, and sweat chloride decreased. BMI also significantly increased.

The main characteristics of the narrative and systematic reviews are shown in Table 5.

**Table 5:** Main features of the narrative and systematic reviews accepted for the review A total of two narrative reviews and four systematic reviews were included in this review. The details of the included studies are summarized in this table. Not reported (NR); Cystic fibrosis (CF); Phenylalanine 508 deletion mutation and an unknown genotype (Phe508del/unknown genotype); Elexacaftor/Tezacaftor/Ivacaftor (ELX/TEZ/IVA); Phenylalanine 508 deletion mutation and a minimal function CFTR mutation (Phe508del/MF); Homozygous for phenylalanine 508 deletion mutation (Phe508del/Phe508del); Forced expiratory volume in one second (FEV1); Cystic fibrosis transmembrane conductance regulator (CFTR); Chloride channel agonist currently only used in clinical trials (VX-659); Elexacaftor (VX-445); Phenylalanine 508 deletion mutation-cystic fibrosis transmembrane conductance regulator (Phe508del-CFTR); Percent predicted forced expiratory volume in one second (ppFEV1); Phenylalanine 508 gene mutation (Phe508); Ivacaftor (IVA); Lumacaftor/Ivacaftor (LUM/IVA); Tezacaftor/Ivacaftor (TEZ/IVA).

First Author, Year	Study Type	Inclusion Criteria	Key points
Tewkesbury, 2021 [4]	Narrative Review	NR	Modulator therapies are likely to improve the course of the CF disease and its management
Marika, 2021 [8]		CF patients with Phe508del/unknown genotype	Treatment of ex vivo models of nasal epithelial cells with ELZ/TEZ/IVA showed excellent responsiveness
Jennifer, 2019 [2]	Systematic Review	Patients aged 12 and above with genotype Phe508del/MF or Phe508del/Phe508del, stable CF disease, and FEV1 % between 40 and 90	Next-generation CFTR correctors VX-659 and VX-445, each in triple combination with tezacaftor and ivacaftor, improve CFTR function in vitro and have shown improvements in phase 2 studies in patients with CF with one or two Phe508del-CFTR alleles.
Andrea, 2020 [11]		Patients aged six years and above, phase 2 and phase 3 trials published from 2005 to 2020	Most studies assessed ppFEV1, safety, and tolerability of ELX/TEZ/IVA as their primary outcome, and all showed clinical improvement
Anielo, 2021 [12]		NR	CFTR modulators have been shown to change the clinical course of the CF in patients heterozygous for Phe508, especially if started at a young age
Dagenais, 2021 [13]		Full manuscripts or conference abstracts of observational studies, case series, and case reports from 2012 to 2020, participants that had a diagnosis of CF that received at least one dose of a CFTR modulator (i.e., IVA, LUM/IVA, TEZ/IVA, or ELX/TEZ/IVA) in the real-world setting, and reported adverse events that occurred while participants were receiving the CFTR modulator.	The types of adverse events reported generally aligned with what has been observed in clinical trials. It is necessary to monitor these effects in people with CF on CFTR modulators in the real-world setting to help better understand potential adverse events and patient characteristics that may be associated with a higher risk of specific adverse events.

Discussion

This section will discuss the pathophysiology of CF, the various mutations that contribute to disease development, and the latest treatment options. The CFTR modulators' efficacy and safety will be touched upon in detail.

Pathophysiology of CF and CFTR Mutations

The CFTR gene found on chromosome seven, codes for the CFTR protein, which is expressed on epithelial cell surfaces. The chloride and bicarbonate channels are the primary responsibilities of the CFTR protein. Consequently, CFTR is a critical regulator of electrolyte transport, and its failure can result in parched liquid layers. This issue puts airways at risk for chronic infection, bronchiectasis, and other problems like pancreatic insufficiency and liver illness [10]. It has been discovered that over two thousand mutations can affect the CFTR gene, as previously stated. The mutations have been categorized into six types based on the molecular defect impairing CFTR function. Phe508del is the most common class II mutation, resulting in incorrect CFTR protein processing and intracellular proteolysis. It has been deduced that individuals homozygous for mutations I-III tend to have less functional CFTR protein, leading to worse phenotypes. On the other hand, patients with class IV-VI mutations frequently have a milder phenotype [4].

CFTR Modulators

Several small molecular compounds have been developed to treat CFTR mutations. Four broad categories exist for these drugs, classified according to their effect on the CFTR protein [10]. Potentiators work by ensuring that the CFTR chloride channel remains open, allowing more chloride ions to pass through. These drugs require the CFTR protein to be present on the cell surface as a prerequisite for proper functioning. CFTR correctors increase CFTR expression at the surface membrane by targeting faulty protein folding [11]. Coupled with ivacaftor, lumacaftor was the first corrector authorized for use due to its ability to restore CFTR function. Stabilizers aim to improve CFTR membrane stability by targeting class II and VI mutations. Lastly, amplifiers stabilize messenger RNA (mRNA) in order to combat CFTR dysfunction. This increases the quantity of CFTR proteins generated and improves translation [12].

Ivacaftor is currently licensed for use in children aged six months and up who have class III to V mutations. In patients homozygous for Phe508del, a combination of ivacaftor and lumacaftor has shown significant clinical improvement. The tezacaftor-ivacaftor combination has also shown compelling improvement, but it is currently only approved for patients above the age of six with a homozygous mutation for Phe508del [5]. Trikafta, a new treatment option including lumacaftor, tezacaftor, and ivacaftor, was recently approved for CF patients aged twelve and up with at least one mutation in the Phe508del gene, regardless of the type of second mutation [13]. This has significantly increased the number of CF patients who can benefit from CFTR modifying treatment. Furthermore, the clinical benefit of elexacaftor-tezacaftor-ivacaftor combination therapy has outperformed other combination therapy alternatives currently accessible to patients, laying the groundwork for a progressive change in the course of CF [14].

Mechanism of Action

In individuals with gating mutations, the CFTR protein enhancer ivacaftor works by increasing chloride current. On the other hand, tezacaftor acts as a corrector to help the mature CFTR protein fold and express itself on the cell surface, thereby enhancing CFTR function in patients with Phe508del mutations. Elexacaftor is a CFTR corrector that operates at a different binding location on the CFTR protein than tezacaftor to improve the CFTR protein's functioning at the cell surface [15]. When administered as a combination, Trikafta enhances the function of the Phe508del mutant CFTR protein on the cell surface, resulting in greater chloride ion transport and amelioration of symptoms [9,16,17].

Dosage and Cost

Trikafta is prepared as a fixed-dose combination tablet containing elexacaftor 100 mg, tezacaftor 50 mg, ivacaftor 75 mg, and individual ivacaftor 150 mg tablets. Every morning, patients must take two combination tablets. For the evening dose, roughly 12 hours later, one ivacaftor 150-mg tablet should be taken. Both doses during the day must be taken alongside a fat-containing meal to maximize effectiveness. If a dose is missed, caution must be taken to avoid double-dosing. At approximately $300,000 per patient per year, Trikafta can be an extremely costly medication for patients. However, in the long run, the revolutionary benefits of this medication far outweigh the cost [18].

Clinical Trials Conducted To Assess the Efficacy and Safety of Trikafta

The efficacy and safety of the Trikafta triple therapy in CF patients have been assessed by three randomized, double-blind phase three clinical trials [3,19,20]. Each trial meticulously studied the drug combination's pharmacokinetics, efficacy, safety, and clinical outcomes following administration [19]. Succeeding the trial period, both studies concluded that the triple therapy significantly improved pulmonary function and disease progression in patients with CF, which led to the Food and Drug Administration (FDA) approval of Trikafta in 2019 [20]. The details of the trials have been summarized in Table 6.

**Table 6:** Clinical trials summary Randomized controlled trial (RCT); Percentage predicted forced expiratory volume in 1 s (ppFEV1); Phe508del/Phe508del (F/F); Sweat chloride (sweat Cl-); Cystic fibrosis quality of life-revised, respiratory domain (CFQ-R RD); Phe508del/minimal function (F/ MF); Body mass index (BMI); Pulmonary exacerbation (PEx); Lung clearance index (LCI-).

First Author, Year	Phase of RCT	Study Duration	Population	Genotype	N	Outcomes
Harry GM, 2019 [3]	III	Eight weeks - Four weeks tezacaftor/ivacaftor run-in - Four weeks trial	Children 12 years or older with 40-90% ppFEV1 and stable disease during the screening period	F/F	n = 52 (tezacaftor, ivacaftor)	Δ ppFEV1: +0.4 Δ Sweat Cl-: +1.7 Δ CFQ-R RD: -1.4
					n = 55 (elexacaftor, tezacaftor, ivacaftor)	Δ ppFEV1: 10.4 Δ Sweat Cl-: -43.4 Δ CFQ-R RD: +16.0
P.G. Middleton, 2019 [6]	III	24 weeks	Children 12 years or older with 40-90% ppFEV1 and stable disease during screening period	F/MF	n = 203 (placebo)	Δ ppFEV1 at 4 weeks: -0.2 Δ ppFEV1 at 24 weeks: -0.4 Δ Sweat Cl-: -0.4 Δ CFQ-R RD: -2.7 Δ BMI: +0.09
					n = 200 (elexacaftor, tezacaftor, ivacaftor)	Δ ppFEV1 at 4 weeks: +13.6 Δ ppFEV1 at 24 weeks: +13.9 Δ Sweat Cl-: -42.2 Δ CFQ-R RD: +17.5 Δ BMI: +1.13 Rate Ratio PEx: 0.37
Edith T., 2021 [7]	III	26 weeks - Two weeks pharmacokinetics study - 24 weeks open label study	Children aged six to eleven years	F/MF	n = 37 (elexacaftor, tezacaftor, ivacaftor)	Δ ppFEV1: + 9.1 Δ CFQ-R RD: + 6.9 Δ LCI2.5: -1.72 Δ Sweat Cl-: -55.1
				F/F	n = 29 (elexacaftor, tezacaftor, ivacaftor)	Δ ppFEV1: + 11.2 Δ CFQ-R RD: + 7.0 Δ LCI2.5: -1.64 Δ Sweat Cl-: -70.4

Clinical Outcomes With CFTR Modulators

Given that declining lung function and pulmonary exacerbations are the leading causes of death in CF patients, all trials documented patient improvement based on these parameters. Furthermore, because pulmonary function tests are easily measurable and quantifiable, they have become the primary outcome of interest in clinical trials [4]. Elexacaftor/tezacaftor/ivacaftor improved forced expiratory volume in one second (FEV1) by 14 points in Phe508del heterozygotes at 24 weeks compared to placebo and by 10 points in homozygotes at four weeks, over tezacaftor/ivacaftor. Moreover, among Phe508del heterozygotes, the triple combination resulted in an impressive 20-point drop in the CF quality of life-revised respiratory domain (CFQ-R RD) score and a 63 percent lower annual rate of respiratory exacerbations compared to placebo [10]. Observational studies of these drugs in daily practice are becoming more common, which is encouraging. A decrease in the number of CF patients on the waiting list for lung transplantation has been documented in case studies [18].

Clinical trial evidence has shown that modulator therapy works not only on the respiratory system. Studies have shown a substantial fall in sweat chloride levels, a biomarker of CFTR protein quality [18]. This may not have significant therapeutic implications, but it suggests that patients can reduce sodium chloride supplementation, usually prescribed for individuals who demonstrate excessive sweating. Furthermore, effective CFTR regulation significantly impacts nutritional and pancreatic health. Elexacaftor/tezacaftor/ivacaftor triple therapy increased body mass index (BMI) and weight in both Phe508del heterozygotes and homozygotes in phase three trials. These findings are promising, given that dietary conditions significantly impact CF outcomes [21]. Additionally, pancreatic damage develops early in life in those with CF, necessitating the use of additional pancreatic enzymes to prevent malabsorption difficulties. Studies done on modulators in children under the age of five suggest that lumacaftor/ivacaftor may improve pancreatic exocrine function in Phe508del homozygous individuals [22]. There are yet to be comparable reports about Trikafta use.

Adverse Effects

The safety of CFTR modulators has been promising thus far [3,11,14]. These medications have been well-tolerated in general, albeit the short interval since FDA approval must be considered. Upper respiratory tract infections, headache, rash, and diarrhea were the most common adverse events in phase three trials. These effects were seen in both the treatment and placebo groups, with more side effects exhibited by the placebo group. 10 percent of the individuals reported rashes, which were more prevalent in the treatment group. The rash can be localized or diffuse and appears papular. Pruritus may accompany it, which can be ameliorated with antihistamines. The rash cleared away in most patients within a few weeks, permitting the continuation of the medication [14]. Multiforme-type urticaria response to Trikafta therapy is explained in a case report published in 2021. The patient presented with swelling of the hands and feet, low-grade fever, and other constitutional symptoms. The drug was discontinued in this patient, following which the rash cleared up [23].

Elevated liver enzymes were found in the safety and efficacy studies of elexacaftor-tezacaftor-ivacaftor, necessitating quarterly blood testing for the first year and annual blood tests afterward. Liver function tests should be tested at baseline, every three months for the first year of treatment, and then once a year after that for patients starting treatment with elexacaftor-tezacaftor-ivacaftor. Treatment should be stopped if aspartate aminotransferase (AST) or alanine aminotransferase (ALT) levels exceed five times the upper limit of normal or if AST or ALT levels exceed three times the upper limit of normal, with bilirubin levels exceeding two times the upper limit of normal. Once abnormal liver function test values have been resolved, a risk-benefit ratio must be assessed before treatment resumes [18,24-26]. In patients homozygous for Phe508del mutations, elexacaftor-tezacaftor-ivacaftor revealed no serious adverse events and no consequences that led to cessation when compared to tezacaftor-ivacaftor. Both experimental groups experienced similar adverse effects, which were mostly resolved by the end of the four-week study [17,25].

Limitations 

Introducing CFTR modulators to the market has had a massive, positive impact on the clinical outcomes of CF patients. However, the meticulous inclusion criteria in RCTs inevitably lead to low representativeness of standard clinical practice. Owing to this, the question of whether these drugs have significant effects in severely symptomatic subgroups of patients that failed to meet the inclusion criteria still stands. Correspondingly, there is a lack of data for patients on the other end of the spectrum, with very mild or absent respiratory involvement. More studies in these areas would be beneficial for clinicians to promptly treat CF patients in daily practice. Another niche area regarding Trikafta therapy is its effectiveness in patients with rare mutations. As it is now approved only for patients with at least one Phe508del mutation, it would be highly informative to have RCTs conducted in patients with other mutations as well. Lastly, the long-term costs and benefits of the triple combination therapy should be assessed over time, perhaps with observational studies. Real-life reports should be gathered and analyzed to infer the cumulative impact of this new drug on not only lung function, but also co-morbidities and mortality.

## Conclusions

In summary, the development of triple therapy has undoubtedly revolutionized the treatment of CF patients. Larger groups of patients have been given a chance at better standards of life, owing to Trikafta. With very few reported side effects, the future of this medication looks promising. However, as new treatments become available, their long-term safety must be assessed for healthcare providers to treat patients effectively. Although the potential adverse events of these medications have been explored in clinical trials, data from real-life experiences with CF patients using CFTR modulators should be shared to establish a more comprehensive conclusion. Furthermore, there remains a sizeable minority of patients who do not qualify for/cannot obtain these medications, which provides future research objectives for CF researchers.

## References

[1] Zaher A, ElSaygh J, Elsori D, ElSaygh H, Sanni A (2021). A review of Trikafta: triple cystic fibrosis transmembrane conductance regulator (CFTR) modulator therapy. Cureus.

[2] Taylor-Cousar JL, Mall MA, Ramsey BW (2019). Clinical development of triple-combination CFTR modulators for cystic fibrosis patients with one or two F508del alleles. ERJ Open Res.

[3] Heijerman HG, McKone EF, Downey DG (2019). Efficacy and safety of the elexacaftor plus tezacaftor plus ivacaftor combination regimen in people with cystic fibrosis homozygous for the F508del mutation: a double-blind, randomised, phase 3 trial. Lancet.

[4] Tewkesbury DH, Robey RC, Barry PJ (2021). Progress in precision medicine in cystic fibrosis: a focus on CFTR modulator therapy. Breathe (Sheff).

[5] Ridley K, Condren M (2020). Elexacaftor-tezacaftor-ivacaftor: the first triple-combination cystic fibrosis transmembrane conductance regulator modulating therapy. J Pediatr Pharmacol Ther.

[6] Middleton PG, Mall MA, Dřevínek P (2019). Elexacaftor-tezacaftor-ivacaftor for cystic fibrosis with a single Phe508del allele. N Engl J Med.

[7] Zemanick ET, Taylor-Cousar JL, Davies J (2021). A phase 3 open-label study of elexacaftor/tezacaftor/ivacaftor in children 6 through 11 years of age with cystic fibrosis and at least one F508del allele. Am J Respir Crit Care Med.

[8] Comegna M, Terlizzi V, Salvatore D (2021). Elexacaftor-tezacaftor-ivacaftor therapy for cystic fibrosis patients with the F508del/unknown genotype. Antibiotics (Basel).

[9] Page MJ, McKenzie JE, Bossuyt PM (2021). The PRISMA 2020 statement: an updated guideline for reporting systematic reviews. Int J Surg.

[10] Nichols DP, Paynter AC, Heltshe SL (2022). Clinical effectiveness of elexacaftor/tezacaftor/ivacaftor in people with cystic fibrosis: a clinical trial. Am J Respir Crit Care Med.

[11] Gramegna A, Contarini M, Aliberti S, Casciaro R, Blasi F, Castellani C (2020). From ivacaftor to triple combination: a systematic review of efficacy and safety of CFTR modulators in people with cystic fibrosis. Int J Mol Sci.

[12] Meoli A, Fainardi V, Deolmi M (2021). State of the art on approved cystic fibrosis transmembrane conductance regulator (CFTR) modulators and triple-combination therapy. Pharmaceuticals (Basel).

[13] Dagenais RV, Su VC, Quon BS (2020). Real-world safety of CFTR modulators in the treatment of cystic fibrosis: a systematic review. J Clin Med.

[14] Southern KW, Patel S, Sinha IP, Nevitt SJ (2018). Correctors (specific therapies for class II CFTR mutations) for cystic fibrosis. Cochrane Database Syst Rev.

[15] Mall MA, Mayer-Hamblett N, Rowe SM (2020). Cystic fibrosis: emergence of highly effective targeted therapeutics and potential clinical implications. Am J Respir Crit Care Med.

[16] Barry PJ, Taylor-Cousar JL (2021). Triple combination cystic fibrosis transmembrane conductance regulator modulator therapy in the real world - opportunities and challenges. Curr Opin Pulm Med.

[17] Muilwijk D, Bierlaagh M, van Mourik P (2021). Prediction of real-world long-term outcomes of people with CF homozygous for the F508del mutation treated with CFTR modulators. J Pers Med.

[18] Joshi D, Ehrhardt A, Hong JS, Sorscher EJ (2019). Cystic fibrosis precision therapeutics: emerging considerations. Pediatr Pulmonol.

[19] Bell SC, Mall MA, Gutierrez H (2020). The future of cystic fibrosis care: a global perspective. Lancet Respir Med.

[20] Yan Z, McCray PB Jr, Engelhardt JF (2019). Advances in gene therapy for cystic fibrosis lung disease. Hum Mol Genet.

[21] Taylor-Cousar JL, Munck A, McKone EF (2017). Tezacaftor-ivacaftor in patients with cystic fibrosis homozygous for Phe508del. N Engl J Med.

[22] Bear CE (2020). A therapy for most with cystic fibrosis. Cell.

[23] Stashower J, Carr P, Miller V, Zlotoff B (2021). Novel reaction to new cystic fibrosis medication Trikafta. Clin Case Rep.

[24] Naehrig S, Chao CM, Naehrlich L (2017). Cystic fibrosis. Dtsch Arztebl Int.

[25] Shteinberg M, Taylor-Cousar JL (2020). Impact of CFTR modulator use on outcomes in people with severe cystic fibrosis lung disease. Eur Respir Rev.

[26] Ponzano S, Nigrelli G, Fregonese L (2018). A European regulatory perspective on cystic fibrosis: current treatments, trends in drug development and translational challenges for CFTR modulators. Eur Respir Rev.

